# When Dad’s Stress Gets under Kid’s Skin—Impacts of Stress on Germline Cargo and Embryonic Development

**DOI:** 10.3390/biom13121750

**Published:** 2023-12-06

**Authors:** Miriam Kretschmer, Vincent Fischer, Katharina Gapp

**Affiliations:** 1 Laboratory of Epigenetics and Neuroendocrinology, Department of Health Sciences and Technology, Institute for Neuroscience, ETH Zürich, 8057 Zürich, Switzerland; miriam.kretschmer@hest.ethz.ch (M.K.); vincent.fischer@hest.ethz.ch (V.F.); 2 Neuroscience Center Zurich, ETH Zürich and University of Zürich, 8057 Zürich, Switzerland

**Keywords:** epigenetic inheritance, stress, sperm, early embryo, development, intergenerational transmission

## Abstract

Multiple lines of evidence suggest that paternal psychological stress contributes to an increased prevalence of neuropsychiatric and metabolic diseases in the progeny. While altered paternal care certainly plays a role in such transmitted disease risk, molecular factors in the germline might additionally be at play in humans. This is supported by findings on changes to the molecular make up of germ cells and suggests an epigenetic component in transmission. Several rodent studies demonstrate the correlation between paternal stress induced changes in epigenetic modifications and offspring phenotypic alterations, yet some intriguing cases also start to show mechanistic links in between sperm and the early embryo. In this review, we summarise efforts to understand the mechanism of intergenerational transmission from sperm to the early embryo. In particular, we highlight how stress alters epigenetic modifications in sperm and discuss the potential for these modifications to propagate modified molecular trajectories in the early embryo to give rise to aberrant phenotypes in adult offspring.

## 1. Epigenetic Inheritance

Pervasive psychological stress imposes an escalating burden on healthcare and society [1], as it emerges as a significant risk factor for the development of neuropsychiatric and neurophysiological conditions, many of which exhibit a hereditary element [2]. Nevertheless, the inheritance of these conditions cannot be fully accounted for by Mendelian inheritance of gene variants. Pioneering research has revealed that irrespective of genetic predisposition, posttraumatic stress disorder (PTSD) exhibits a heightened prevalence among the offspring of Holocaust survivors with PTSD [3,4]. The legacy of trauma is, in part, perpetuated by behaviour. Yet simultaneously the environmental, in this case traumatic stress, might directly impact the physiology and psychology of their descendants. Thus, the inheritance of complex diseases potentially encompasses the transmission of non-genetic information to the next generation, a concept commonly referred to as epigenetic inheritance. This process involves the transmission of environmentally modified molecular factors to the succeeding generation through parental gametes, extending beyond the realm of genes [5]. Importantly, these information carriers must not only be transferred via parental gametes to the embryo but also maintain their altered state, potentially resulting in enduring changes in the embryo’s epigenomic makeup (as reviewed in [6]).

Nevertheless, exploring the impact of environmental influences on the non-genetic inheritance of complex diseases presents extensive challenges. Human studies have primarily relied on descriptive correlations and associations, where the confounding effects of socio-economic factors and the intricacies of the overall complex human environment remain beyond control. This limits the potential for mechanistic investigations to establish causal links between information carriers and the inheritance of diseases.

Animal models enable rigorous control over environmental and genetic confounding factors, facilitating detailed mechanistic examinations. Due to their similarity to humans and the feasibility of conducting transgenerational studies, rodents have been extensively studied, laying the groundwork for investigating the epigenetic inheritance of stress-related traits. Historically, the initial evidence supporting epigenetic inheritance emerged from studies conducted using a mouse model with variant epialleles in the agouti locus [7,8,9]. Variations in gene activity appeared to evade epigenetic reprogramming or be reinstated after incomplete erasure as they passed through the female germline, thereby transmitting epigenetic information to their descendants. This initial evidence was mechanistically substantiated for sperm RNA-mediated inheritance by the group of Minoo Rassoulzadegan in 2006. Animals with a heterozygous mutation in the *Kit* gene exhibited a white tail tip, a trait also observed in their wild-type offspring. They postulated that altered *Kit* RNA levels and the sizes of parental testes might be responsible. To confirm this, they injected purified total sperm RNA or testis RNA from *Kit* mutant mice into wild-type zygotes, resulting in offspring displaying white tail tips [9]. Utilising the same method, other research groups confirmed that sperm RNA species are conveyed to the oocyte [10,11]. Subsequently, injections into wild-type zygotes became a standard technique for investigating the mechanisms of epigenetic inheritance. After an initial surge in studies on RNA-mediated epigenetic inheritance, various techniques and tools have since been developed to examine a plethora of epigenetic marks that could serve as potential information carriers, including DNA methylation, histone post-translational modifications (PTMs), and chromatin structure, among others.

The majority of the literature addressing the mechanistic aspects of epigenetic inheritance focuses on the sperm epigenome and paternal transmission of environmental exposures. This bias is likely due to the relatively smaller number of potential confounding factors when compared to maternal–offspring effects. Not only do gestational signals play a role, but post-gestational maternal care, such as nutrition and nurturing, can also influence the offspring’s phenotype. While these factors, along with the limited availability of oocytes, complicate the study of maternal intergenerational effects, epigenetic modifications in oocytes have been studied, and their potential role in epigenetic inheritance has been examined elsewhere [12,13].

In the realm of paternal transmission, the most extensively studied epigenetic information carrier is DNA methylation. A multitude of models reporting altered DNA methylation patterns in sperm in the context of intergenerational effects have been reported, particularly in the context of endocrine disruptors and toxic substances [14,15,16,17]. In the following sections, we will review the effects of stress on altered paternal DNA methylation and also point out that the causal implication of DNA methylation is still lacking. In this review, we explore how the epigenetic modifications of paternal gametes can be influenced by stress. We also delve into the epigenetic modifiers along the developmental trajectories of embryos and discuss the evidence indicating that alterations in sperm can affect these modifiers.

## 2. Vertical Information Carriers of Epigenetic Inheritance in the Paternal Germline?

Over the past decade, substantial advancements have been made in unravelling some mechanistic aspects responsible for the epigenetic inheritance of stress-induced phenotypes. This progress can be attributed to the swift development of innovative technological protocols employing next-generation sequencing techniques. Through these advancements, researchers have pinpointed potential candidates that become altered due to stress and have the potential to influence this inheritance. Their investigations have revolved around understanding which epigenetic changes occur in sperm in response to environmental stressors and whether these modified alterations exert an influence on the subsequent generation. In this review, we examine the epigenetic modifications in sperm that have been observed to undergo changes in response to stress. Subsequently, we explore how these modifications might impact embryonic development.

### 2.1. Non-Coding RNAs

Non-coding RNAs, which constitute the majority of the transcribed genome, play a pivotal role in regulating gene expression and the resulting transcripts [18,19]. Interestingly, mature sperm, characterised by transcriptional silence and low levels of mRNA, contain a diverse array of non-coding RNA species. This includes microRNAs (miRNAs), which are small non-coding RNAs measuring 21–25 nucleotides (nt), known for their role in gene expression regulation by targeting mRNA transcripts [20]. These miRNAs interact with the 3′ UTR of mRNAs, recruiting the RNA-induced silencing complex to inhibit or degrade target transcripts [21]. miRNAs have been found to be necessary for spermatogenesis [22].

Likewise, P-element-Induced WImpy testis (PIWI)-interacting RNAs (piRNAs) play a crucial role during spermatogenesis. These small non-coding RNAs, measuring 26–31 nt, are associated with the regulation of gene expression by interacting with the PIWI subfamily of Argonaute (Ago) proteins [23]. They are implicated in stabilising the germline genome through the methylation of transposable elements at the prepachytene stages [23] and contribute to post-translational mRNA cleavage at the pachytene and post-meiotic stages [24,25].

Intriguingly, sperm RNA does not primarily consist of miRNAs and piRNAs, but instead, the majority of small non-coding RNAs in sperm are derived from transfer RNA (tRNA) fragments (tRFs) [26]. tRFs are relatively larger, measuring 29–34 nt, and originate from the 5′ and 3′ ends of tRNAs. A recent preprint confirmed the prevalence of 5′ derived tRFs, as reported in earlier studies [26,27], yet showed more balanced levels of both 5′ and 3′ derived tRFs [28], owing to a novel RNA cloning protocol known as Ordered Two-Template Relay [29]. tRFs are known for their role in somatic cells where they associate with Ago proteins to cleave sequence-matched targets [30], while their function during spermatogenesis remains undiscovered. They have, however, been proposed to regulate MERVLs in the early embryo, retrotransposable elements from a murine endogenous retrovirus [27]. MERVLs induce the stage-specific regulation of transcription of genes specific to totipotency during ZGA. This is achieved through the transcription of MERVLs using their long terminal repeat as promoters and the subsequent activation of neighbouring genes [31,32,33,34].

In contrast, long non-coding RNAs (lncRNAs), exceeding 200 nt in size, have been implicated in spermatogenesis by regulating gene expression [35]. lncRNAs can be categorised into bidirectional, sense, antisense, intronic, and intergenic lncRNAs, and they also play a role in gene regulation by influencing chromatin structure [36]. Furthermore, circular RNAs (circRNAs), ranging from 500 to 4000 nt in size, are formed through back splicing, a unique form of splicing. In this process, the 5′ and 3′ ends of pre-mRNA are covalently linked, creating highly stable circular non-coding RNAs. These circRNAs are particularly abundant in mouse testes [37,38]. They are increasingly produced during the late pachytene spermatocyte stages until the elongating spermatid stage, and some of them serve as templates for peptide translation during the final stages of spermatogenesis [39]. Worth mentioning is that circRNAs have also been described as functioning as miRNA sponges and/or as protein complex stabilisers in other contexts [40,41].

The composition of RNA in sperm significantly differs from the RNA found in somatic cells and can undergo qualitative or quantitative alterations due to environmental influences [42,43,44]. However, the question of whether sperm non-coding RNA is a remnant of spermatogenesis or if it is acquired from the surrounding somatic tissue and environment remains a matter of debate. RNA changes may occur in somatic cells and subsequently be transported to the testis or epididymis via the bloodstream [45,46]. This notion was supported by a study using a model for early life stress that uses unpredictable maternal separation and maternal stress (MSUS), where alterations in sperm miRNAs were reflected in blood serum [47]. Alternatively, it was suggested that sperm RNAs may be taken up during epididymal transit via extracellular vesicles (EVs). The group of Oliver Rando showed that a group of tRFs in sperm, that were altered upon feeding a low-protein diet, were unaltered in testes but did show changes in the epididymal tissue, reasoning that altered tRFs must have been taken up during epididymal transit [27]. By co-incubating sperm with epididymal epithelial exosomes in a follow-up study, they could for the most part recover the same altered tRF profile in sperm, concluding that tRFs in sperm are acquired during epididymal transit via EVs [27]. This was later confirmed in a follow-up study assaying small RNA dynamics across spermatogenesis using SLAM-seq, a sequencing method based on the metabolic labelling of RNA [48]. This sentiment was corroborated by a different group where changes in sperm miRNA were much more prevalent 12 weeks after four weeks of chronic randomised psychological stress as opposed to one week after, concluding that they had been taken up during epididymal transit, which requires two weeks in mouse [49]. Tracking EVs using a novel labelling approach based on an HA-tagged tetraspanin endogenous to EVs, the authors showed EV content to be taken up and present within sperm heads [50]. Conversely, a recent study proposed that the majority of non-coding RNA in sperm is acquired from cytoplasmic droplets, transient organelles storing RNA specifically in testicular and epididymal sperm [51]. Comparing the small RNA content of earlier spermatogenic cells, the study suggested that small RNAs in sperm are derived from nuclear RNAs of late spermatids during spermatogenesis. This study further proposed that rRNA-derived small RNA (rsRNA) constitutes the largest small RNA fraction, which had not been previously reported due to the removal of rsRNA before library construction in earlier studies [51].

Importantly, sperm RNA composition has been suggested to impact the health of the offspring [52]. Consequently, numerous studies have attempted to understand how sperm RNAs can be influenced by the environment and their origin. In this review, we summarise the findings regarding how stress can affect sperm RNAs, while a broader overview of how environmental factors influence sperm RNAs can be found in other sources [5,53,54,55,56,57]. Alterations in sperm non-coding RNA due to stress have been reported in studies as recent as a decade ago, thanks to advancements in next-generation sequencing allowing for genome-wide RNA sequencing (Table 1).

Among the early findings, Gapp et al. observed changes in sperm small RNA composition when exposing mice to early life stress in the first two weeks following birth [47]. Males subjected to MSUS exhibited altered sperm RNA content, particularly an increased expression of 43 miRNAs, five of which were also confirmed to be altered in the brain and serum through quantitative PCR (qPCR). Furthermore, cluster 110 of piRNAs was down-regulated in the sperm of these mice [47]. One of the miRNAs altered due to MSUS, miR-375, was subsequently confirmed to be changed in a different stress model. Male mice at four weeks or 14 weeks of age showed increased expression levels for nine miRNAs after 42 days of exposure to chronic variable stress during puberty or adulthood. This stress included various random chronic stressors such as constant light for 36 h, exposure to fox odour for 15 min, overnight exposure to a novel object, restraint in a 50 mL conical tube for 15 min, multiple cage changes, overnight exposure to 100 dB white noise, and saturated bedding overnight [44]. Similarly, Wang et al. exposed males aged eight weeks to three mild stressors daily for five weeks, including a wet cage, food deprivation, restraint, stroboscopic illumination with 150 flashes per minute, inversion of the light-dark cycle, a 45° tilted cage, and loud noise ranging from 90 to 105 dB. This led to changes in the expression of 19 miRNAs (18 up-regulated and 1 down-regulated) and 24 piRNAs (6 up-regulated and 18 down-regulated) in their sperm as observed through RNA-seq and qPCR [67]. Altered sperm miRNAs were also reported in a study using a different stressor, namely, chronic social instability. At the age of four weeks, male mice were housed in a weekly randomised composition of mouse cages for seven weeks [62]. This resulted in the downregulation of two miRNAs, as confirmed via qPCR. Notably, this study found no changes in miR-375, which had been previously reported to be altered in two independent studies by Gapp et al. [47] and Rodgers et al. [44]. These discrepancies in observed sperm RNA changes may be attributed to differences in experimental design, including mouse breed, stressor, and detection methods using RNA-seq or qPCR.

In another study employing the MSUS model, changes were also observed in long RNAs in sperm from exposed males. In addition to small non-coding RNAs, hundreds of lncRNAs and mRNAs were altered upon chronic stress exposure [63]. This finding was corroborated in a study using another stressor, chronic social defeat stress. C57/BL6J males were exposed daily to novel aggressive CD1 males for 10 min and then separated by a plexiglass barrier to allow for sensory contact. This resulted in significant alterations in lncRNAs, with differences between animals classified as susceptible or resilient to stress based on the social interaction test [65].

Stress has also been shown to affect sperm RNA composition when acute activation of the glucocorticoid receptor is induced, through high-dosage dexamethasone injections, a glucocorticoid receptor agonist [64]. Two weeks after injection, 22 miRNAs (18 up-regulated and 4 down-regulated), 44 tRFs (17 up-regulated and 27 down-regulated) and ribosomal RNA fragments exhibited changes in sperm from the cauda epididymis. This study also detected increased expression levels of two circRNAs, a class that, as mentioned above, was previously described in mouse sperm but had not been assessed in the context of environmental perturbations. Interestingly, the circRNA changes were only detectable in cauda sperm but not in caput sperm [37,38].

In summary, the studies described above demonstrate the impact of stress on the composition of sperm non-coding RNA. However, inconsistencies in reporting and differences in RNA classes make it challenging to establish a comprehensive understanding of RNA alterations under stress. Furthermore, comparing results across studies is hindered by variations in stressors, mouse strains, and the use of RNA-seq versus qPCR for detection. Lastly, the intricate composition of RNA in sperm and its changes upon stress necessitates an inquiry into the source of this sperm RNA.

### 2.2. DNA Methylation

Another epimodification that has been extensively explored in the context of epigenetic inheritance is DNA methylation. DNA methylation occurs at CpG dinucleotides in mice, catalysed by DNA methyltransferases (DNMTs), and is a major gene expression regulator [69,70]. It is known for its remarkable stability as an epigenetic mark. It plays a crucial role in genetic imprinting, a process through which parental influences impact offspring gene expression through allele-specific methylation. A wealth of research has reviewed this topic extensively [71,72,73,74].

DNA methylation undergoes two major reprogramming events over an individual’s lifetime, specifically during the pre-implantation embryo stage and gametogenesis. In the zygote, approximately 30% to 70% of regions undergo demethylation as a result of DNA replication and conversion of 5-methylcytosine to 5-hydroxymethylcytosine, facilitated by ten-eleven translocation dioxygenases (TETs) [6,75,76]. At imprinted genes and other certain loci, DNA methylation remains unchanged. Subsequent to this initial reprogramming, DNA methylation undergoes another round of erasure at approximately E10.5–E13.5 in primordial germ cells, leading to the retention of only 7–14% of parental DNA methylation [75,77,78,79,80,81]. DNA methylation is subsequently re-established around E15.5, resulting in a fully established germ cell profile in pachytene spermatocytes at birth.

Despite the relatively low retention of paternal DNA methylation, the influence of environmental factors, such as stress, on the methylation pattern in sperm has been examined (Table 1). For instance, studies using the MSUS model reported changes in methylation levels at the promoters of several candidate genes in the sperm of the F0 generation (three up-regulated, two down-regulated, and two unaltered) [58,60,82]. Similarly, increased methylation at the promoter region of a target gene in sperm was observed in a study using chronic restraint stress, which involved immobilising adult mice in a 50 mL falcon tube for 2 h daily for 14 days [61].

Kong et al. exposed adolescent four-week-old mice to chronic restraint stress, albeit for 8 h daily for 8 weeks in a micro cage. While global methylation was only assessed in unexposed female offspring, they reported differential methylation levels at the promoters of eight candidate genes (five up-regulated and three down-regulated) in the sperm of exposed fathers [66]. Sperm methylation changes induced by stress were only recently investigated at a genome-wide level. In this research, mice were subjected to immobilisation in a 50 mL falcon tube for 2 h daily for 90 days, beginning at 3 weeks of age. The sperm displayed 24,427 differentially methylated regions, with about 50% located within promoters or gene bodies [68].

While genomic imprinting is a well-established and well-studied phenomenon, the mechanism by which stress induces DNA methylation changes in sperm remains largely unknown. It is worth noting that to date, no study has addressed the causal implication of a stress-induced DNA methylation change on offspring phenotypes. In contrast to the above-mentioned single study that assessed methylation genome-wide in sperm following a stress exposure, other environmental perturbations have been extensively studied regarding their effects on DNA methylation [15,83].

### 2.3. Histone Modifications

Histone octamers, comprising a tetramer composed of H3–H4 histones and two H2A–H2B heterodimers, serve as the architectural foundation for chromatin subunits known as nucleosomes, wherein DNA is coiled around them spanning a length of 147 base pairs (bp) [84]. In higher eukaryotic organisms, a multitude of histone isoforms exist for each histone type, their occurrence being contingent upon the particular cell type and the stage of the cell cycle [85,86]. In addition to the previously mentioned isoforms, histones can display variability arising from PTMs occurring along their peptide tails. This spectrum of modifications includes established small molecules and extensively studied PTMs, including but not limited to methylation, acetylation, and phosphorylation. Furthermore, these modifications extend to more sizable molecular moieties such as, mostly associated with neurotransmitters, serotonin and dopamine, contributing to the complexity of histone diversity [87,88,89]. The diverse spectrum of histone marks is intricately linked to a multitude of effects on chromatin dynamics. The nature of these marks dictates the recruitment of distinct proteins, colloquially classified as readers, writers, and erasers, each with distinct roles [90]. These recruited proteins can function as chromatin remodelers, orchestrating processes that lead to either the compaction or relaxation of chromatin structure [91]. As a consequence, in a simplified way, chromatin undergoes alterations in its accessibility to the transcription machinery, consequently exerting an impact on gene expression dynamics [92]. To provide a more comprehensive example, it is noteworthy that diverse histone marks can be linked with distinct stages of transcription [93].

When examining male germ cells and their final mature product, a distinct and highly specialised cellular type emerges. The chromatin within mature sperm exhibits a remarkable degree of condensation, a feat achieved through the process of histone-to-protamine replacement. This coordinated transformation involves the displacement of histones by protamines, resulting in a remarkably compacted chromatin structure. The proportion of histones that persist throughout this replacement process varies, encompassing a range of 1–5% in mice [94,95]. The achievement of successful spermatogenesis in fertile sperm relies upon the careful arrangement of histone variants and histones harbouring post-translational modifications, a subject that has been extensively explored in in-depth reviews elsewhere [96,97]. Histone variants which were shown to be important for spermatogenesis are, besides others, TH2A and TH2B (testis-specific variants of the respective histones). Additionally, the histone variants H2AL1/2 and H2BL2 are not only present during spermatogenesis but also in mature spermatozoa [98]. Furthermore, the replication-independent H3 variant, H3.3, is important for successful maturation [99,100] and it was detected in mature sperm, as well [101].

As mentioned above, despite the extensive process of histone replacement, nucleosomes carrying distinct histone marks were detected within regions linked to regulatory elements (such as enhancers and promoters) of genome loci that are also of importance in the early embryo or mouse embryonic stem cells (mESCs) [102]. Additionally, such nucleosomes with specific histone marks can also be detected at loci characterised by repetitive elements [103]. Indeed, investigations have revealed a convergence in the distribution of H3Kme1 and H3K27ac within enhancer regions of mESCs and sperm [104].

Evidence from multiple studies has illuminated the susceptibility of the histone code within sperm to alterations. The impact of stress on histone PTMs in sperm has not been explored besides at a global level in caput epididymis sperm [49]. Here, animals experienced chronic variable stress during their adolescence and young adulthood. PTM analysis with mass spectrometry showed that the main driver of PTM composition, namely time (one versus 11 weeks after stress), was disrupted by the chronic stress paradigm. Nevertheless, numerous investigations have delved into the impact of specialised dietary regimens and their effect on sperm PTMs, such as high-fat or alcohol consumption, on the levels of histone markers H3K4me3 and H3K9me2 [105,106,107,108]. Notably, H3K4me3 is frequently localised at the 5′ terminus of actively transcribed genes [109], and its perturbation has been linked to RNA polymerase pausing and decelerated elongation [110].

In summary, evidence of stress affecting histone PTMs in mouse sperm is currently lacking. However, the plenitude of evidence for other environmental perturbations affecting histone PTM composition suggests the potential for stress to also induce alterations in histone PTMs in sperm.

### 2.4. Chromatin Structure

We are also starting to appreciate the biological functionality of the three-dimensional arrangement of chromatin itself [111]. This renders sperm particularly captivating for investigation due to its remarkably compacted chromatin structure. Contemporary exploration of this three-dimensional configuration is becoming feasible through innovative sequencing methodologies like Hi-C that capture the spatial proximity of various genome sequences to one another [112].

This approach has elucidated the categorisation of genomic segments into distinct compartments. These compartments are emblematic of distinct genomic landscapes: one category of “A” compartments is characterised by active transcriptional genes and the presence of histone marks indicative of active chromatin, while another category of “B” compartments encompasses regions housing inactive genes and repressive chromatin marks [113]. Within the broader A and B compartments, further subdivisions can be envisioned, so-called topologically associating domains (TADs). These domains offer a finer resolution of genomic interactions, underscoring the connections between gene sequences. Typically, regions residing within a TAD exhibit a markedly heightened probability of interacting with one another in contrast to regions situated beyond the borders of the TAD [114].

Fascinatingly, analyses of mouse sperm chromatin utilising Hi-C have unveiled congruent 3D structural patterns exhibiting remarkable similarity to other cell types, akin to the division observed in other cellular types like fibroblasts and mESCs [102,115]. Furthermore, TADs identified within sperm showcased a reduced count of 1856 in contrast to the 2590 found in fibroblasts, albeit marked by a pronounced enlargement in size. When comparing bioinformatically determined TADs between fibroblasts and sperm, it was shown that multiple smaller TADs in the former can be detected as a single larger TAD in the latter [115]. Moreover, sperm chromatin exhibits a propensity for extensive long-range interactions spanning distances of 50 to 150 Mega base pairs (Mb). This observation stands in contrast to shorter-range interactions, which occur less frequently compared to those observed in fibroblasts [115]. Applying increased sequencing depth, a study reported a greater prevalence of interactions between TADs as opposed to interactions within a TAD [116]. This phenomenon seems paradoxical, given that TADs are traditionally defined as genomic regions with an elevated number of interactions within in comparison to regions beyond their bounds (McArthur & Capra, 2021). Additionally, the sperm genome harbours a noteworthy number of extra-long-range interactions, spanning distances greater than 4 Mb, as well as inter-chromosomal interactions [115]. The prevalence of extended interactions between distant regions within the sperm chromatin is plausible, considering the compacted nature of its chromatin. This high condensation effectively brings together disparate genome regions in close proximity, potentially accounting for the increased interaction between distant long-range regions as compared to other cell types.

The impact of stress on the three-dimensional organization of sperm remains a subject that, to our current knowledge, lacks studies using NGS technology. However, existing research exploring DNA damage has indicated that physiological stress, besides other environmental factors, does indeed lead to oxidative stress, fragmentation, and an overall reduction in fertility rate and sperm count [117,118,119]. These effects, in turn, can most likely influence the three-dimensional structure of chromatin. While direct evidence from mouse sperm is absent, it is worth noting that radiation-induced DNA damage, although not specifically in mouse cells, has been associated with changes in the three-dimensional organization of topologically associating domains (TADs) in human fibroblasts. This perturbation resulted in increased segregation between TADs, as observed in a study involving radiation-induced DNA damage in human fibroblasts [120].

Taken together, while comprehensive studies directly addressing the impact of stress on the 3D organization of mouse sperm via NGS technology are currently lacking, existing research on DNA damage and related factors does provide suggestive insights into potential alterations in chromatin structure due to stress.

### 2.5. Transcription Factors

Transcription factors (TFs) are proteins that either bind individually or form complexes with other TFs to attach to specific TF motifs on the DNA, thereby controlling gene expression and the organization of 3D chromatin [121]. Specific TFs, such as CTCF, Fox1, ERα, and AR, have been identified as occupying the sperm genome through ChIP sequencing. In the case of other TFs, their presence has been inferred from chromatin accessibility data [102,104].

The study of how environmental perturbations can affect TFs in mouse sperm is limited, likely due to the technical challenges associated with working with highly compacted sperm chromatin. Nevertheless, certain transcription factors can access compacted chromatin as pioneering factors and subsequently recruit other factors and transcription machinery to specific regions. In contrast, others rely on their target regions being accessible. This intricate interplay between TFs, DNA methylation, and chromatin organisation complicates the investigation of individual TFs’ functions and may necessitate more comprehensive approaches to understanding how paternal stress-acquired traits can be transmitted.

## 3. Epigenetic Regulators in the Early Embryo—How Do Stress-Induced Changes in the Paternal Epigenome Impact Them?

Deciphering the complexities of the regulation of embryonic development has been a multi-decade endeavour. To ensure the transition from a single omnipotent cell to a highly differentiated multicellular organism, specific transcriptional programs must be executed in a well-timed and distinct manner. Additionally, the epigenetic alterations in the paternal and maternal pronuclei (PN) must originate from the parental gametes, from which the embryo arises. Any disruptions in these modifications could potentially impact early embryonic transcriptional regulations and set forth an altered regulatory path that may influence the health or disease predisposition of the offspring. In this context, we consider findings on the role of epigenetic modifications in regulating pre-implantation embryos and summarise the evidence supporting a causal connection between alterations in the paternal epigenome and pre-implantation embryos. We also explore how stress-induced changes in paternal gametes can impact the developmental trajectory of the embryo.

### 3.1. Non-Coding RNA

Among the many species of non-coding RNAs, the function of lncRNAs in early embryonic development has been characterised most, specifically their involvement in dosage compensation and genomic imprinting. For example, *Xist* was the first discovered lncRNA essential for X-chromosome dosage compensation. The deletion of *Xist* via homologous recombination led to the demise of female mice in early embryogenesis, resulting from an extra-embryonic phenotype caused by the presence of two active X-chromosomes [122]. The precise mechanisms by which lncRNAs like *Xist* regulate dosage compensation have been extensively reviewed elsewhere [123,124,125,126,127,128]. Apart from their involvement in dosage compensation and imprinting, lncRNAs in early embryos have been found to impact cell fate decisions. For instance, lncRNA *Carm1* acts as an epigenetic marker for inner cell mass (ICM) of the blastocyst at the 2/4-cell stage, influencing the determination of cell fate for trophectoderm and ICM [129,130,131,132].

miRNAs make up a relatively small proportion of non-coding small RNAs in pre-implantation embryos [133]. Initially, their expression levels are low and increase rapidly after the 2-cell stage [134]. They were previously thought to be essential for clearing maternal mRNAs during the maternal-to-zygotic transition [135]. However, a later study demonstrated their dispensability for maternal mRNA clearance by knocking out the gene for the microprocessor DGCR8, a cofactor of DROSHA important for processing primary miRNA transcripts into miRNA precursors [136]. Notably, endogenous siRNAs and miRNAs generated by mirtrons were not affected in their function, suggesting alternative DGCR8/DROSHA-independent miRNA mechanisms during pre-implantation development [134,136]. However, the knockout of DICER, which processes precursor miRNAs into mature miRNAs, in the oocyte resulted in miRNA depletion in the embryo and failure of the first cell division due to disorganized spindle formation [135,137]. Further downstream in the miRNA pathway, maternal AGO2 was knocked out in another [138]. AGO is the catalytic component of the RNA-induced complex, which, guided by miRNA, cleaves target mRNA. Knockout of maternal AGO2 led to the stabilisation of target mRNAs and the failure of the embryo to develop past the 2-cell stage [138]. These findings on maternal miRNAs were complemented by a study investigating paternal RNAs. The aberrant phenotypes observed in DICER or DROSHA knockout embryos were rescued by injecting control sperm miRNA and endo-siRNA [139]. With the onset of transcription during the zygotic genome activation (ZGA), miRNAs from the highly conserved miR-430/427/302 family start to accumulate in the 2-cell stage embryo, playing a major role in the regulation of mRNA transcripts of genes in signalling pathways for stem cell pluripotency maintenance, including *Stat3* and *Tet3*, among others [133,134,135,140,141]. miRNAs of the miR-290–295 cluster are also present initially but increase rapidly at the 4-cell stage.

While the primary role attributed to piRNAs has been described during spermatogenesis (reviewed in [142]), they have also been identified in the 2-cell stage [27] and pre-implantation embryos [134,139]. piRNAs are mainly derived from the oocyte and are mostly cleared during pre-implantation development [133,143]. In mice, oocytes contain less piRNAs than in other mammals. The repression of transposons is taken over by endogenous siRNAs [142,144,145,146]. The role of maternal piRNAs in embryonic development has not been clearly defined.

In addition to piRNAs, pre-implantation embryos also contain maternally derived endogenous siRNAs, which, like piRNAs, are cleared during the maternal-to-zygotic transition (Ohnishi et al., 2010). These siRNAs repress retrotransposons in the oocyte and persist in the zygote until cleared [144,145,146].

Advances in single-cell and small RNA sequencing, such as Ordered Two-Template Relay [29], will likely further aid in unravelling the map of small non-coding RNAs and their dynamics during embryonic development. Nevertheless, studies involving interventions to dissect the roles of individual non-coding RNAs have contributed to our understanding of how they regulate early embryonic expression. While this was rarely done in pre-implantation embryos due to technical constraints, many studies manipulated non-coding RNAs in sperm and examined how these changes in the paternal epigenome affect embryonic development. Early experiments involving sperm RNA injection into fertilised oocytes date back to 2006 [9]. In a seminal study, animals with a heterozygous mutation for the *Kit* gene were used to produce wild-type offspring. Surprisingly, the offspring exhibited the parental phenotype of white tail tips, which was attributed to abnormal *Kit* RNA levels and sizes in the parental testes. Purifying parental total sperm or testis RNA and injecting it into wild-type zygotes produced offspring with the white tail tip phenotype. This study concluded that paternal RNA alone was sufficient to transmit the phenotype [9].

In subsequent years, RNA injections into naïve zygotes were employed to investigate whether stress-induced changes in sperm RNA could affect the developmental trajectories of the resulting offspring. Gapp et al. used RNA microinjections to demonstrate the epigenetic inheritance of a stress-induced phenotype for the first time [47]. Mice subjected to early life stress in the form of MSUS exhibited a predisposition to risk-taking behaviour, despair, and insulin hypersensitivity, in addition to alterations in sperm miRNA and piRNA profiles. This phenotype was reproduced in the offspring of naïve zygotes injected with purified total RNA from exposed males. The study concluded that sperm RNA alone was sufficient to transmit the phenotype, although the contribution of piRNAs could not be ruled out [47]. This idea was further supported by a follow-up study using the MSUS model, in which Gapp et al. demonstrated changes in lncRNAs and mRNA in sperm from exposed males. Many of these long RNAs correlated with the altered abundance in the zygote resulting from the mating of MSUS males with naive females. To interrogate a function of paternal lncRNAs, Gapp et al. purified sperm RNA from exposed males and injected either the small (<200 nt) or long (>200 nt) RNA fraction into naïve zygotes. Offspring resulting from the injection of small RNA only replicated the behavioural despair phenotype, while those resulting from the injection of the long RNA fraction displayed partial replication of the risk-taking behaviour, behavioural despair, and altered glucose response [63]. Only the injection of both small and long RNA fractions led to a complete phenocopy in the offspring [47]. Similarly, another group reported changes in sperm miRNA in response to chronic variable stress during a different susceptibility window, namely adolescence [44], and the microinjection of the affected sperm miRNAs into naïve zygotes resulted in a blunted stress response in the resulting offspring (Rodgers et al., 2015). The offspring also exhibited reduced gene expression levels in the paraventricular nucleus of the brain, including genes associated with extracellular matrix terms crucial for the HPA axis [59]. The transmission of stress-induced effects through sperm small RNAs was elegantly rescued in a recent study by Wang et al. Exposing male mice to unpredictable mild stress resulted in a depression-like phenotype and an altered sperm miRNA profile, and injecting purified sperm RNA from exposed males reproduced the phenotype in the offspring. Co-injecting antisense strands of the affected miRNAs into the zygote rescued the offspring phenotype [67].

**Figure 1:**
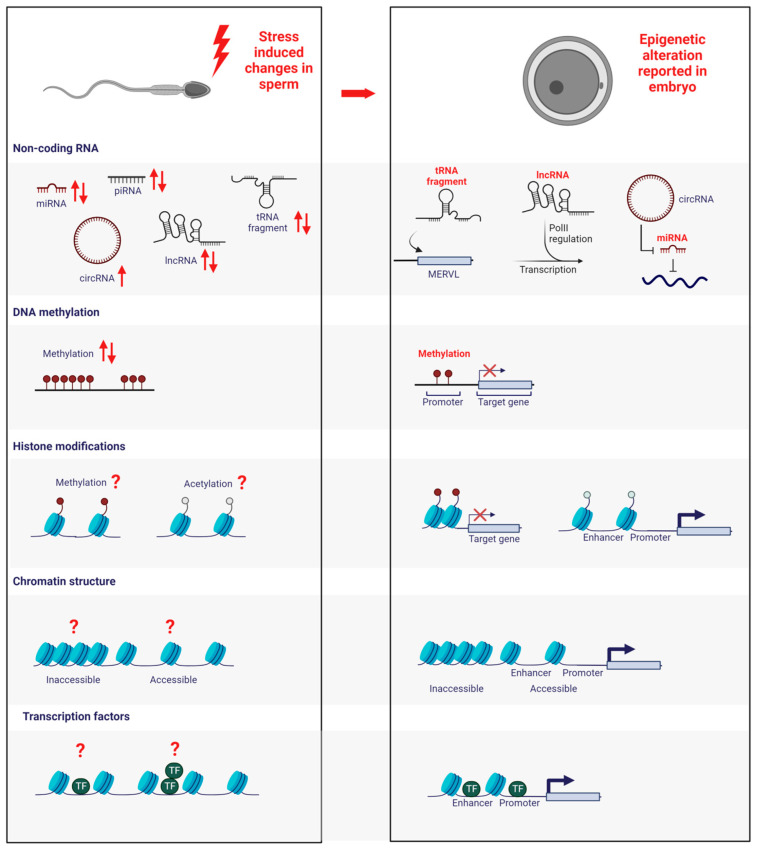
Stress induced changes of the sperm epigenomes and implications for embryonic gene regulation. Left side: Stress induces changes in abundance and prevalence of non-coding RNAs and DNA methylation in sperm, as indicated by red arrows. Alterations in histone modifications, chromatin structure, and transcription factors in sperm have been reported using other environmental exposures, yet remain unclear in the context of stress, as represented by question marks. Flash symbolizes environmental stress insult. Right side: Gene regulatory functions during early embryonic development have been reported for all these epigenetic modifications. Among them, tRNA fragments, miRNAs, lncRNAs and DNA methylation have been reported to be altered in the embryo upon paternal stress exposure, as indicated by red font. Figure was created 24 November 2023 using BioRender.com (accessed on 24 November 2023).

Although it is evident that stress-induced paternal phenotypes can be transmitted through altered sperm RNA content, the mechanisms by which these RNAs affect the embryo have yet to be fully explored. In the context of a dietary intervention, Sharma et al. did assess the consequences of altered sperm tRf by injection them into naive fertilised oocytes to assess the consequences on early embryonic gene expression. They observed the de-repression of genes regulated by MERVL in the resulting embryos [27]. While the mechanisms of action in the embryo of altered sperm RNA content are unclear, it is, however, established that sperm RNA aberrations cause alterations in the offspring (Figure 1). A proof-of-principle study investigated this by employing RNase enzymes to abolish the sperm RNA content [147]. Intracytoplasmic injection of treated sperm resulted in reduced blastocyst formation and live birth rates. This effect could be rescued by the co-injection of RNA purified from wild-type sperm. The authors concluded that paternal RNA is essential for early embryonic development [147].

The consequences of altered sperm RNA following paternal stress were partially addressed in a study by Gapp et al., which employed a pharmacological intervention involving Dex exposure, leading to significant changes in the sperm RNA profile of exposed male mice [64]. While the paternal phenotype was not assessed, the offspring exhibited metabolic alterations, including increased BMI and altered glucose tolerance. Small RNA sequencing of 2-cell embryos resulting from Dex- or vehicle-injected mice revealed the downregulation of several tRFs from six genomic locations. Smart-seq RNA sequencing of these embryos unveiled a shift in genes associated with late 2-cell stage functions for the Dex-injected offspring, including genes known to be involved in early embryonic development, such as *Rbbp7* and *Pcl1* [64]. Only two other studies reported changes in the embryonic epigenome reflecting stress-induced alterations in sperm. The first being the above-mentioned study on lncRNA and mRNA by Gapp et al., where MSUS-induced alterations in sperm lncRNAs were reflected in zygotic lncRNAs [63]. In the second, male mice exposed to chronic social instability stress displayed changes in sperm miRNA, which were reflected in the pre-implantation embryo [62]. However, the latter study did not assess offspring phenotype following RNA injection, so it remains unclear whether the altered miRNAs transmit a stress-induced phenotype.

In summary, the transmission of stress-induced phenotypes through altered sperm RNA payload is well-established, but the precise mechanism of action in the embryo remains largely unknown. Two main questions require further investigation: 1. On which targets do paternal non-coding RNAs exert their function? 2. How long do paternal non-coding RNAs persist in the pre-implantation embryo? Answering the first question relies on the examination of the immediate effects on early embryonic gene expression. Given that significant transcription programs only commence after the first cleavage of the zygote [148], detecting subtle changes in gene expression at the zygote stage may be technically challenging. DNA methylation and histone occupancy, including changes in DNA methylation and histone occupancy, may be employed to unravel the impact of paternal RNA on the early embryo. To answer the second question, tracking paternal non-coding RNAs will be essential. This could be achieved by either visual or quantitative methods like LC-MS or sequencing in combination with the labelling of RNA at fertilization and in the pre-implantation embryo.

### 3.2. DNA Methylation

DNA methylation represents one of the major regulators of gene expression during embryogenesis, as it supplies molecular memory, ensuring the preservation of hereditary information [149,150,151]. In adult somatic cells, methylation patterns tend to remain more stable. However, during embryonic development, two critical reprogramming events shape global methylation patterns, occurring in the zygote and during gametogenesis around E10.5–E13.5.

The initial reprogramming event initiates with DNA demethylation of the paternal PN, followed by a decrease in global methylation levels in the zygote until the blastocyst stage [73,152,153,154,155,156,157,158]. This hypomethylated state ensures pluripotency and precise regulation of future differentiation [159,160,161]. After implantation, methylation levels rise, establishing cell lineage differentiation [157,158,162]. The co-expression of DNMT3 and TET enzymes significantly contributes to genome-wide de novo methylation [163,164]. The second reprogramming event occurs during the period of E10.5–E13.5 when primordial germ cells (PGCs) originating from the epiblast undergo demethylation and establish a sex-specific methylation pattern during gametogenesis [165].

These two waves of demethylation are crucial for ensuring the faithful commitment to transcriptional programs during embryonic development, facilitating the shift from pluripotency to cell lineage differentiation. To achieve this, methylation must be maintained during these waves for specific genes such as imprinted genes [166,167], retrotransposons [168,169], Rhox genes [170], and germline genes [74,171]. By maintaining methylation levels at these regions, the distinct parental DNA methylomes can influence the [71,72,73,74,172]. Based on intriguing cases, such as at the agouti locus, the idea of epialleles emerged, that is, genes with an imprinted-like manner escape reprogramming. In mice carrying the mutation, the endogenous retrovirus intracisternal A particle (IAP) retrotransposed and inserted upstream the agouti gene, resulting in an alternative promoter that is epigenetically very unstable and variably methylated within one organism, resulting in mosaic coat colours of various shades of yellow. The phenotype was passed on maternally to some extent [7]. However, it is worth noting that work by Ferguson-Smith’s group suggests that the memory of the parental epiallelic methylation state, as seen in genetically identical mice with the agouti viable yellow locus is more of an exception than the rule [173]. Looking at IAP-containing loci in general, they found that very few were acting as promoters, and their parental methylation status did not predetermine the methylation level in the offspring. Hence, the authors concluded that their findings are challenging the idea of a generalised epigenetic inheritance of epigenetic states [173]. Furthermore, a follow-up study by the same group investigated genes with a reported parent of origin bias—hence, a kind of imprinted expression pattern. Applying stringent analysis parameters, they found that most of the reported cases likely were false positives due to genetic background or a neighbouring truly imprinted gene. Hence, the list of imprinted-like genes is also far less extensive than previously thought [173].

The maintenance of methylation in retrotransposons and imprinted genes throughout embryonic development is thought to primarily depend on DNMT1, not DNMT3A or DNMT3B, as only a knockout of *Dnmt1* led to globally reduced methylation levels across all genomic loci at E8.5. Double knockout of DNMT3A and DNMT3B reduced overall methylation levels, but not at transposable elements, which retained their hypermethylated state (Dahlet et al., 2020). These knockout studies revealed that, apart from imprinted and germline regions and transposable elements, only a few regions of the paternal methylome are retained [77]. Although allele-specific transcription was observed before ZGA at some loci other than imprinted regions and retrotransposons, DNA methylation levels at these loci have not been determined in these studies [34,174]. In addition, a recent study compared multiple published methylation, chromatin accessibility, and RNA-seq datasets [175]. The authors found that TF-bound sites remained hypomethylated during the methylation reprogramming events during development, in contrast to dynamically de- and remethylated unoccupied sites [175]. Among these, maintaining the hypomethylated state of LINE-1 retrotransposons is crucial. LINE-1s are another class of transposable elements that are highly expressed in the pre-implantation embryo [176], peaking during ZGA [177], and are presumed to play a pivotal role in gene regulation networks [178], mainly by maintaining the permissive state of chromatin in the early embryo after fertilization and during ZGA [177,179]. Both LINE-1s and IAPs are then silenced by H3K9me3 between the 8-cell and the blastocyst stage [177,180,181]. Artificial repression of LINE-1s reduces chromatin accessibility, while aberrant prolonged expression prevents chromatin compaction [182]. The maintenance of hypomethylation of LINE-1s during reprogramming is achieved via repressive histone marks installed by the chromatin assembly factor 1. In the absence of this histone chaperone, the initiation of silencing H3K9me3 fails, and this results in an aberrant upregulation of LINE-1, yielding lethality at the morula stage [180].

Retaining methylation at sites other than transposable elements potentially provides an opportunity to carry over epigenetic information to the zygote. This is especially the case if the retained methylation is within functionally relevant loci. Therefore, the impact of environmental influences, such as stress, on the methylation pattern has been investigated, paying particular attention to transcriptional start sites and, generally, promoters. For instance, in studies using the MSUS model, methylation was altered in the sperm of the F0 generation [58,60,82]. Animals exposed to MSUS displayed depression-like behaviour, which was also observed in their non-exposed offspring. The researchers linked this to aberrant methylation levels in the promoters of candidate genes in sperm, including transcriptional regulator methyl CpG-binding protein 2 and stress hormone receptor corticotropin-releasing factor receptor 2 [58]. These changes were also observable in offspring sperm [58], brain [60,82] and blood serum [183]. A similar association between paternal sperm and offspring brain methylation level was also found in studies involving paternal adolescent restraint-stressed subjects [66]. Although the paternal phenotype was not assessed, the unexposed offspring displayed reduced anxiety-like behaviour compared to offspring sired by control males. This was reflected in altered gene expression in the offspring’s hippocampus (734 up-regulated and 338 down-regulated), involving genes related to inflammation and neurodevelopmental disorders. Of the eight candidate genes reported to be differentially methylated in the sperm of exposed males, six displayed alterations in the same direction in the hippocampus of unexposed offspring [66]. Another study investigating a stress-induced metabolic phenotype reported methylation changes in the sperm of the parental generation that were not detected in the offspring’s brain but in the liver [61]. Exposure to chronic restraint stress led to increased blood glucose levels in exposed males and hypermethylation in the promoter for *Sfmbt2* in sperm. Unexposed offspring similarly exhibited increased hepatic gluconeogenesis and increased expression of a gene involved in gluconeogenesis, phosphoenolpyruvate carboxykinase. This was due to the repressing miRNA for this gene being encoded in the *Sfmbt* gene, which was hypermethylated in the offspring’s liver. The study concluded that the paternal methylome could transmit the stress-induced metabolic phenotype to the offspring [61]. How alterations in sperm methylation levels translate into affected methylation of the same locus in a specific tissue of the offspring remains elusive. To fill this gap, the dissection of all intermediate steps during development would likely be required. Zheng et al. pioneered efforts toward achieving this objective in their study investigating stress-induced global methylation changes in sperm from exposed males as well as in the embryos sired by these males [68]. In that study, 11.36% of the differentially methylated regions in sperm were present in the direct offspring, and 0.48% even persisted in the F2 offspring. These regions were associated with genes related to the stress response. Importantly, methylation at these regions was erased and re-established, evading embryonic reprogramming. This resulted in the replication of the metabolic phenotype in the form of elevated blood glucose levels and a reduction in anxiety-like behaviour, along with increased risk-taking behaviour, observed in the fathers being replicated in the unexposed offspring [68].

Further investigations into the mechanisms of DNA methylation-transmitted inheritance of paternally acquired traits are needed. Conventional epigenome editing in the compacted sperm chromatin appears challenging should the fertilization capacity of sperm remain. Furthermore, causally linking DNA methylation to gene expression, especially when (de)methylated regions regulate distal genes, remains tricky. A recent study overcame these obstacles and provided a first proof of concept for the transmission of an aberrant parental methylation status using epigenetically edited mESCs [184]. Targeted GC methylation was established in mESCs in the GC islands of two metabolism-related genes, Ankyrin domain 6 and the low-density lipoprotein receptor, silencing these genes. This was achieved through CRISPR/Cas9-mediated insertion of CpG-free DNA, which was subsequently de novo methylated and then excited to leave only the epigenetically altered state without affecting the base pair sequence itself persistently. Edited mESCs were then microinjected into 8-cell stage embryos, generating chimeric mice with up to 99% of the edited genome. These mice displayed increased body weight and elevated serum leptin levels, a phenotype also seen in Ankyrin domain 6 knockout mice. The acquired methylation and phenotype persisted until the F4 generation but were erased and re-established during embryonic development [184], similar to what had been demonstrated in another region [68]. The mechanism responsible for maintaining the installed mark remains unknown, and further research is needed to determine if this mechanism occurs naturally.

Aberrant methylation marks in several offspring organs have been linked to stress-induced changes in the paternal methylome [58,60,61,66,68]. However, it cannot be ruled out that the observed phenotypes were due to indirect effects. The methylome is intricately connected with covalent and non-covalent chromatin organization, meaning that multiple epigenetic marks are involved in regulating a single genomic region. These marks could be located at different loci. To definitively attribute these marks to genomic effects, labelling and tracking individual marks or employing epigenetic editing [184] to dissect the underlying mechanisms would be necessary.

### 3.3. Histone Modifications

Histone modifications play a crucial role in governing interactions between transcriptional regulators and chromatin during pre-implantation embryogenesis [185,186]. Reprogramming events during early embryo development result in the removal of most histone modifications, but similar to DNA methylation, some marks persist at imprinted regions and a few other loci [187,188]. Understanding the temporal dynamics of histone modifications during pre-implantation development has been a focus of numerous studies.

Due to the limited presence of retained histones in sperm, di- and tri-methylation histone marks on H3K9, H4, and H3K27me3 are exclusively detected in the maternal PN of the zygote [189,190,191,192,193,194]. Methylation marks on H3K4 are initially detected in both PN but rapidly diminish in the paternal PN until major ZGA re-establishes them [107,189,193,195]. Once firmly established at the 2-cell stage, H3K4me3 exhibits dynamic changes at promoter regions, overall increasing until the first lineage decision [189,192,193]. The downregulation of H3K4me3 due to maternal demethylase KDM5B overexpression results in defective genome silencing in the oocyte [193]. Loss of H3K4 methylation leads to a compromised minor ZGA activation, which is primarily driven by paternal allele-specific transcription, resulting in developmental delays and reduced survival rates [196]. This effect was achieved by injecting K4 with a mutated methylation site before fertilization, leading to altered H3K4 methylation levels, especially in the paternal PN. The study concluded that the reduced global methylation levels of the paternal PN caused alterations in the minor ZGA, inducing growth retardation [196]. Non-canonical H3K4me3, represented by broader ChIP-seq peaks at partially methylated sites, is present on the maternal genome but is removed in the late 2-cell stage [193]. Similarly, H3K64 is solely found in the maternal PN, but it is drastically reduced by the two-cell stage [197,198]. While initially only detected in the maternal PN, H3K9me3 maintains allele-specific imbalance and is increasingly established with progressing development [181]. It is largely installed at long terminal reads of retrotransposons, including MERVL, after the 4-cell stage, coinciding with the reduced expression of the retroelements [180,181]. In the post-implantation embryo, H3K9me3 marks the first lineage differentiation [181]. In contrast to H3K9me3 dynamics, H3K27me3 is maintained until the morula stage, specifically at the maternal allele, after which it decreases [199,200]. This mark is highly enriched in promoters and is associated with the regulation of imprinted genes and select retroelements [181]. Like H3K9me3, H3K27me3 is associated with the regulation of enhancer activation and lineage-specific genes at the post-implantation state [190]. At imprinted loci, including Xist, H3K27me3 works in concert with H2AK11ub1 in dependence on maternal polycomb repressive complexes [201]. Acute H2AK11ub1 depletion in the zygote causes premature ZGA and embryonic arrest, while H3K27me3 imprinting maintenance remains unaffected [201]. Mutation of the histone variant H3.3K27 de-represses paternal heterochromatin, resulting in dysfunctional chromosome segregation and developmental arrest [202]. In contrast to their trimethylated counterparts, which are only detected in the maternal PN, the paternal PN displays mono-methylation marks at H3K9 and H3K27 [203], and H3K4 [196]. Allele-specific studies show that H3.3 is further retained in the paternal genome during the PN stage of embryo development [204].

This retention of parentally derived marks, as well as the differential presence of histone PTMs between maternal and paternal PN, implies a function for these inherited marks. Environmental perturbations could affect histone PTM occupancy and frequency in the gametes and remain as a signal to the developing embryo. This possibility was explored in a few studies, yet to a limited degree within the context of stress exposure. Lismer et al., in particular, demonstrated that certain regions of H3K4me3, modified by diets rich in folic acid and high fat, manifest changes that are also detectable in the resulting early embryo generated from such [107]. Additionally, the histone mark H3K4me3 has been observed to coincide with regions exhibiting tissue-specific patterns, as exemplified by its presence in distinct patterns within tissues such as the placenta and testes [102,107]. A study investigating the function of epididymal extracellular vesicles following stress also assessed the impact on global levels of histone PTMs in sperm. In addition to alterations in the non-coding RNA profile of extracellular vesicles isolated from caput sperm of chronically stressed mice, discernible disparities in the composition of histone marks were unveiled between stressed and non-stressed subjects, as demonstrated through the use of mass spectrometry. While lacking sequence-specific information, this study illustrated that how stress could potentially influence the arrangement of histone marks, thus acting as a conduit for conveying information within developing mammalian sperm [49].

Histone modifications display a distinct pattern between the parent-of-origin alleles. The differences, however, might be overestimated due to limitations in capture-based sequencing methods. Reduced material from the PN and possible differences in target abundance limit the current protocols. Further, suitable antibodies are essential but not available for all targets. Improved sequencing methods like low-input CUT&TAG [205] or the improved ChIP-seq protocol for sperm cells and embryos [206] and advancements in epigenome editing might help decipher occupancy and function of paternal histone PTMs in the early embryo. The manipulation of these via environmental exposure suggests that they can transmit acquired phenotypes to the offspring. However, to date, there is no evidence for stress-induced alterations of histone PTMs in sperm. Whether such altered PTMs might evoke alterations in embryonic development is implied by studies using other environmental perturbations but remains to be shown in the context of stress.

### 3.4. Chromatin Structure

The effective development of embryos relies on the precise regulation of chromatin structure. This modulation is essential for rendering chromatin accessible to transcription factors that play a pivotal role in determining the timing of genome activation and governing transcription. During the initial stages of mouse embryo development, the accessibility of chromatin undergoes dynamic changes.

Due to the contrasting chromatin structures of highly condensed sperm DNA and the more accessible oocyte DNA, parental chromatin must undergo extensive reprogramming upon fertilization. Initially, the sperm head begins to decondense upon entry into the oocyte, and the parental genomes remain separated as two distinct PN within the zygote [207]. Protamines in the paternal PN undergo active phosphorylation by SRKP1, are subsequently dismissed by NPM2 [208], and are replaced by H3.3 through the action of HIRA, assembling nucleosomes de novo [196,202,209,210,211]. While the PN are still separate, their chromatin accessibility is reduced, particularly at cis-regulatory elements, until they reach the required accessibility levels for the activation of all genes necessary for major ZGA [34,212,213]. During this phase, before and after ZGA and following protamine removal, both PN exhibit comparable and synchronized levels of chromatin accessibility, with a few cases of allele-specific accessible chromatin and transcription [34,174]. These accessible regions, in this permissive state, are associated with pluripotency regulators and have predicted binding sites for transcription factors involved in development, such as NFYA, NANOG, OCT4, SOX17, and AP2Y [154,214]. During and after ZGA, the increased chromatin permissiveness of regions distal to transcription start sites correlates with the activity of enhancer elements [34,154]. The rapid dynamics of chromatin states in the embryo are enabled by chromatin remodelers such as SWI/SNF [215,216,217,218,219,220], CHD chromatin remodelling complexes [221,222,223], and ISW/NURF [224,225,226]. The mode of action of these remodelers, dependent on ATP, has been reviewed elsewhere [227,228].

It is plausible to assume that environmental factors that induce altered levels of chromatin remodelers could have profound consequences on early embryonic remodelling and transcription. Studies in pre-implantation embryos have demonstrated lethal effects in knockout models of several chromatin remodellers. Notably, knockouts of subunits of the SWI/SNF complex result in early embryonic lethality [215,218], peri-implantation lethality [216,219], and lethality in later developmental stages [217,220]. Knocking out subunits of CHD chromatin remodelling complexes led to pre-blastocyst stage developmental arrest [222] and lethality at later stages [221]. Knockouts of subunits of the ISWI/NURF family led to peri-implantation [225] and post-implantation lethality [224]. These knockout studies underscore the essentiality of chromatin remodellers for embryonic survival. Whether these remodellers can be perturbed in gametes by environmental challenges remains to be investigated.

During embryonic development, not only is the chromatin accessibility and the presence of remodellers crucial in ensuring the proper execution of transcriptional programs, but the hierarchical 3D chromatin structure also holds significance. Technical limitations have resulted in limited investigations into the 3D chromatin structure in mouse embryos, though early temporal dynamics have been unveiled [116,154,229]. In the PN, 3D organization remains ambiguous, but paternal chromatin retains separation and distinct compartmentalization during ZGA [229,230,231]. TADs are gradually established in the pre-implantation embryo in a parent-organization-specific and ZGA-independent manner [116,229]. In early 2-cell embryos, Polycomb-associating domains emerge on the maternal allele and deteriorate by the 8-cell stage [232]. Naturally, 3D chromatin organization during early embryonic development goes hand in hand with the reprogramming of other epigenetic modifications, yet the exact underlying molecular mechanisms are yet to be understood. For example, TAD formation has been found to occur during the cell cycle transition rather than at ZGA [116]. Chromatin accessibility and 3D organization determine not only the transcriptional programs during embryonic development but also the reprogramming of epigenetic modifications. Understanding their molecular mode of action is crucial for comprehending the interplay of epigenetic regulatory networks during early embryonic development.

Chromatin accessibility in gametes naturally impacts the expression of mRNAs and their proteins, determining the transcriptome and translatome at the onset of the maternal-to-zygotic transition. A recent study curating available RNA-seq datasets across spermatogenesis and the zygote suggested that sperm mRNAs for epigenetic enzymes, including histone lysine writers and erasers, are translated in the zygote and contribute to chromatin assembly in the embryo [233]. Disturbing the mRNA levels for these enzymes in gametes may result in significant changes in the trajectories of embryonic development. This could be achieved by altering the accessibility of enhancers of genes in the early embryo. Notably, findings by Victor Corces’ group underscore the importance of proper 3D chromatin organization not only in the embryo but also in sperm. They showed that disrupting the chromatin structure in sperm affects the subsequent development of the embryo. By probing pre-implantation embryos with Hi-C sequencing, they demonstrated that CTCF-dependent chromatin organization is maintained in a parent-organization-specific manner until the 8-cell stage [102,104]. Alterations in the paternal chromatin structure, caused by the endocrine disruptor Bisphenol A, resulted in an abnormal metabolic phenotype in the offspring [234]. This was attributed to changes in the accessibility of CTCF binding sites at cis-regulatory elements of the *Fto* gene, which is implicated in obesity. Altered CTCF binding increased the interaction of cis-regulatory elements with two genes relevant to the differentiation of appetite-controlling neurons, *Irx3* and *Irx5*. Importantly, the deletion of the CTCF binding site in *Fto* rescued the phenotype induced by paternal Bisphenol A exposure. The authors concluded that alterations in paternal chromatin structure at *Fto* could result in the same offspring phenotype as genetic variations [234].

Whether such changes in chromatin accessibility in sperm can be induced by stress exposure remains to be investigated. Furthermore, it is unclear whether and how differential accessibility is maintained.

### 3.5. Transcription Factors

Transcription factors play a pivotal role in governing gene expression and 3D chromatin organization [121]. However, research on transcription factors in early embryos has faced challenges in detecting these low-abundance proteins until recent technological advancements.

In the zygote, the paternal PN exhibits higher transcriptional levels and a more transcriptionally permissive state than the maternal PN [235]. Consequently, more TFs occupy the paternal PN compared to the maternal PN [236]. Specifically, TFs identified in the zygote using confocal immunofluorescent microscopy include SP1 and TBP [235,236], OCT4 and ETS1 [237], NFYA [154] and members of the DUX family [238]. While their functions can be inferred from extensive research in mESCs [239,240,241], only a few transcription factors have described roles in the embryo. For instance, NFYA promotes chromatin accessibility [242], and its knockdown in zygotes resulted in significant changes in gene expression during ZGA (83 up-regulated and 297 down-regulated) [154]. YAP1 knockout led to defective gene activation during ZGA, resulting in aberrant cell compaction and TE lineage specification [243], underscoring the essential role of certain transcription factors in ZGA. DUX, which is transiently expressed at the early 2-cell stage, was proposed to drive ZGA [238,244]. Deleting DUX by using CRISPR/Cas9 delayed ZGA in two independent studies but did not prevent some knockout embryos from surviving into adulthood [245,246]. Prolonged DUX expression led to developmental arrest, emphasizing the need for degradation and silencing processes to ensure proper gene regulation in the pre-implantation embryo [246]. Investigation of upstream factors that initiate ZGA in a DUX-dependent or -independent manner revealed developmental-pluripotency-associated genes 2 and 4 as key regulators [247]. Their binding to DUX was stabilized by another transcription factor, ZSCAN4C, as determined by using ChIP-seq. This increased DUX expression and initiated ZGA [247]. Expressed slightly later at the 2- and 4-cell stage, ZSCAN4C induced the expression of the retroelement MERVL [248]. MERVL, unlike most other retrotransposons, is expressed in the 2-cell stage embryo and is involved in gene expression regulation, as described above [249], and chromatin structure reorganization through donation of promoters containing CTCF binding sites [250]. Motif enrichment analysis revealed binding motifs for several transcription factors, including CTCF, NR5A2, TEAD4, GATA4, POU5F1, SOX2, and NANOG, present in the pre-implantation embryo, all of which are crucial during early embryonic development [34,104,251]. However, further research is needed to establish whether the sites are indeed occupied and at what specific time frames during pre-implantation development. More reliable antibodies targeting transcription factors and technological advancements, such as the ultra-low-input adaptation of the CUT&RUN protocol [252], may help this endeveour.

The transcription factors detected in the zygotic pronuclei are either derived from parentally transmitted mRNA or directly transferred as proteins at/around parental chromatin. This suggests that environmental factors could influence the occupancy and availability of transcription factors in gametes. To date, only one study has explored environmental influences on paternal transcription factors and their consequences in the embryo. Jung et al. inferred the inheritance of transcription factors from sperm by comparing TF ChIP sequencing data and TF motif abundance in accessible chromatin of gametes with accessibility data in embryos up to the 8-cell stage [102,104]. They concluded that chromatin organization in the early embryo relies on CTCF, an essential architectural protein that serves as an anchoring point for TADs [253,254] and is involved in transcriptional regulation, chromatin insulation and higher-order chromatin organization [253,255]. The knockout of CTCF results in embryonic lethality due to impaired chromatin structure formation and developmental arrest at the blastocyst stage [251,256,257]. To investigate the potential susceptibility of CTCF-dependent chromatin organization to environmental factors, Jung et al. induced obesity in male mice through Bisphenol A exposure, which was transmitted across multiple generations. However, mice with a mutated CTCF binding site at a specific *Fto* enhancer with altered accessibility, failed to transmit the obesity phenotype induced by bisphenol A, presumably due to the failure to recruit CTCF to an *Fto* enhancer [234].

Given that several transcription factors have been identified in sperm chromatin, and their motifs are present in the embryo genome at the same loci [34,102,104], their potential role in transmitting acquired phenotypes cannot be disregarded. Further experiments, for instance confirming the occupancy of embryonic chromatin by transcription factors through approaches like ChIP or CUT&RUN [206,258] and the transfer of paternal transcription factors from sperm to the embryo will be essential to see whether the case reported for CTCF [234] can be extrapolated to further TFs.

## 4. Stress Sensitivity as a Converging Effect of Distinct Types of Exposures

While in this review, we concentrate on stress as an environmental exposure, other studies involving rodents investigating the inheritance of traits acquired from the environment have reported psychological and behavioural effects in the offspring due to various types of exposures. These exposures induced behaviours in the unexposed progeny, indicating heightened stress sensitivity. It is crucial to emphasize that these studies were conducted using rats, and distinct behavioural assessments were utilised in each investigation. Early on, the group of Michael Skinner demonstrated that exposure to the fungicide vinclozolin resulted in modified sperm DNA methylation patterns in unexposed male offspring [14]. In a subsequent study, they observed altered behaviour and stress response among other phenotypes in the unexposed offspring [259]. Similarly, another study involving exposure to Bisphenol-A reported changes in the social behaviour of unexposed offspring [260]. Parallel effects on offspring were noted in studies involving anesthetics, where exposure to morphine increased anxiety-like behaviour [261], and sevoflurane exposure led to altered stress response [262]. Reports of altered offspring stress response also followed paternal ethanol exposure [263], and increased offspring anxiety was noted in a study involving heroin exposure [264].

Collectively, these studies clearly illustrate a transgenerational effect of the initial pollutant, anesthetic, or drug exposure, resulting in modified psychological phenotypes in the offspring. However, they largely do not elucidate how gametes were molecularly affected by the exposures. Additionally, the intergenerational transmission mechanism and how altered epigenetic modifications might influence the developmental trajectory of the embryo remains to be seen. However, it seems important to consider that other types of exposures—especially those that converge on hormone receptor function—could also modify mental health-related outcomes in offspring.

## 5. Conclusions

We have gathered evidence indicating that stress, as well as broader environmental factors, can exert an influence on the epigenome of sperm. This influence encompasses non-coding RNAs, DNA methylation, post-translational modifications of histones, chromatin structure, and transcription factors. While it is generally assumed that the majority of the sperm epigenome undergoes reprogramming during pre-implantation development, there are instances, particularly in the face of significant disturbances, where reprogramming appears to be circumvented. This becomes apparent as these exposures are linked to observable traits in subsequent generations that were not directly exposed to the stressors. However, the precise mechanisms by which the paternally inherited fraction of the embryonic epigenome evades reprogramming and the factors determining which modifications are retained or re-established following erasure remain unclear. To address how resulting offspring phenotypes come about, it is imperative to unravel the processes occurring in the early embryo that set the course for the emergence of said divergent phenotypes. Improved tools for characterising changes in both the sperm and embryonic epigenomes are essential. Additionally, targeted manipulation is necessary to establish causal relationships. A comprehensive exploration of the epigenome at various levels is warranted. Potential alternative pathways for transmitting stress-related traits should not be omitted, including DNA damage and mutations, copy number variations, components of seminal fluid [265] and, obviously, behavioural transmission [266]. Last but not least, even though not covered in this review, the female germline and maternal exposure cannot be neglected under any circumstances. Crucial to all these efforts is gaining a deep understanding of the molecular processes operating in the early embryo.

## Figures and Tables

**Table 1:** Studies investigating stress-induced changes of the sperm epigenome.

Study	Stressor	Epimodification Affected in Sperm	Epimodification Affected in Offspring (Organ)	Paternal Phenotype		Offspring Phenotype		Causality Checked
				Behavioural	Metabolic	Behavioural	Metabolic	
Franklin et al., 2010 [58]	MSUS	DNA methylation	DNA methylation (sperm)	+	N.A.	+	N.A.	N.A.
Rodgers et al., 2013 [44]	chronic variable stress	miRNA	N.A.	N.A.	N.A.	+	+	N.A.
Gapp et al., 2014 [47]	MSUS	miRNA, tRF, piRNA, DNA methylation	miRNA (serum)	+	+	+	+	RNA injection
Rodgers et al., 2015 [59]	chronic variable stress	miRNA	−	N.A.	N.A.	N.A.	+	RNA injection
Gapp et al., 2016 [60]	MSUS	DNA methylation	DNA methylation (brain)	+	N.A.	+	N.A.	N.A.
Wu et al., 2016 [61]	chronic restraint	DNA methylation	DNA methylation (brain)	N.A.	+	N.A.	+	N.A.
Dickson et al., 2018 [62]	chronic social instability	miRNA	miRNA (embryo)	N.A.	N.A.	N.A.	N.A.	embryonic miRNA
Gapp et al., 2020 [63]	MSUS	lncRNA	lncRNA (zygote)	+	N.A.	+	+	RNA injection
Gapp et al., 2021 [64]	Dexamethasone	miRNA, tRF, rRNA, circRNA	tRF	N.A.	N.A.	N.A.	+	embryonic tRF
Cunningham et al., 2021 [65]	chronic social defeat	lncRNA	N.A.	+	N.A.	+	N.A.	N.A.
Kong et al., 2021 [66]	chronic restraint	DNA methylation	DNA methylation (brain)	−	−	+	N.A.	N.A.
Y. Wang et al., 2021 [67]	chronic variable stress	miRNA, piRNA	−	+	+	+	+	RNA injection, antisense strand RNA injection
X. Zheng et al., 2021 [68]	chronic restraint	DNA methylation	DNA methylation (embryo)	+	+	+	+	N.A.

Epimodification/phenotype were reported to be affected by paternal stress (+) or not to be affected by paternal stress (−). N.A indicates where studies did not report on epimodification, phenotype or causality.
